# Eating Speed, Physical Activity, and Cardiorespiratory Fitness Are Independent Predictors of Metabolic Syndrome in Korean University Students

**DOI:** 10.3390/nu13072420

**Published:** 2021-07-15

**Authors:** Minjeong Kang, Mingyu Joo, Haeryun Hong, Hyunsik Kang

**Affiliations:** College of Sport Science, Sungkyunkwan University, Suwon 16419, Korea; kangmin125@skku.edu (M.K.); jmg0403@naver.com (M.J.); hhr8028@skku.edu (H.H.)

**Keywords:** behavioral risk factors, eating speed, metabolic complications, university students

## Abstract

Background: Little is known regarding the role of eating quickly, physical inactivity, and poor cardiorespiratory fitness (CRF) in assessing the onset of metabolic syndrome (Mets) in Korean young adults. Objectives: This study examined the association between the three risk factors and Mets in 1891 Korean university students (30% female) aged 18–29 years. Methods: Eating speed (slow vs. fast) and physical activity (active vs. inactive) were assessed with a standardized questionnaire. Maximal oxygen uptake as an indicator of CRF was assessed with graded exercise testing. Components of Mets were waist circumference, systolic and diastolic blood pressures, fasting blood glucose (FBG), triglycerides (TG), and high-density lipoprotein cholesterol (HDLC). Results: All the three exposures were positively associated with abdominal obesity, elevated blood pressures, elevated FBG, elevated TG, and decreased HDLC. Logistic regression analysis showed that the odds ratio (OR) of Mets was incremental in the order of physical inactivity (odds ratio, OR = 1.666; 95% confidence interval, CI = 1.024–2.708; *p* = 0.040), fast eating (OR = 1.687; 95% CI = 1.094–2.601; *p* = 0.018), and poor CRF (OR = 5.378; 95% CI = 3.475–8.325; *p* < 0.001). Conclusions: The current findings suggest that a multifaceted intervention targeting at promotion of physical activity and CRF in concert with healthy eating behaviors should be implemented as a preventive strategy against Mets in Korean university students.

## 1. Introduction

Metabolic syndrome (Mets) reflects the clustering of abdominal obesity, elevated blood pressure (BP), elevated blood glucose, elevated triglycerides (TG), and reduced high-density lipoprotein cholesterol (HDLC) and constructs a common risk factor underlying the development of metabolic disorders such as cardiovascular disease [1] and type 2 diabetes [2]. Physical inactivity and poor fitness together with excessive caloric intake are three of the major behavioral risk factors responsible for the onset of Mets in children and adolescents [3,4] and in adults [5,6].

Lack of physical activity is a well-known determinant of Mets [7,8]. Cardiorespiratory fitness (CRF) refers to the oxygen-carrying capacity of the cardiorespiratory systems to the skeletal muscles for energy production of sustained physical activity, and poor CRF is another independent determinant of Mets [5,9]. By contrast, the protective effects of being physically active and promoting CRF with respect to Mets have been systematically reviewed and well summarized in previous meta-analysis studies [10,11].

Findings of previous research showed that eating quickly is a modifiable risk factor responsible for overweightness and obesity [12,13], poor glycemic control [14], elevated TG [15], and Mets [16] in different populations. By contrast, eating slowly was associated with healthy body weight [17] and less energy intake [18]. Taken together, it is likely that all the three risk factors, including physical inactivity, poor CRF, and eating quickly, may either individually or collectively increase one’s susceptibility to the onset of Mets and other metabolic disorders.

To the best of our knowledge, however, no previous study has examined the adverse effects of eating quickly, physical inactivity, and poor CRF on the onset of Mets in Korean college students. Investigating the association between those risk factors and Mets may provide essential information to support its prevention, especially given that the risk for Mets increases with aging [19]. This study investigated the relationships of the three behavioral risk factors with Mets in Korean university students.

## 2. Materials and Methods

### 2.1. Subjects

**Figure 1:**
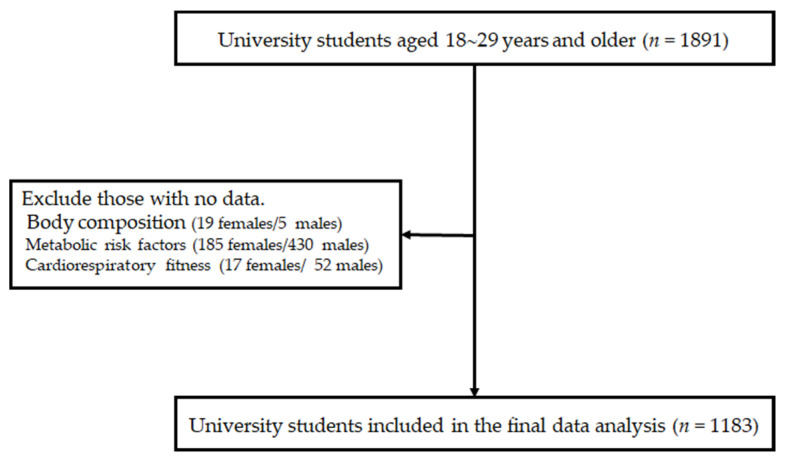
Flow of eligible study participants.

We initially invited 1891 university students (30% female) who registered for the lecture “exercise and health” during the 2017 to 2018 and 2018 to 2019 academic years at Sungkyunwan University, Suwon, Republic of Korea. All participants aged 18–29 years were apparently healthy, had no diagnosed chronic diseases (i.e., diabetes, hypertension, dyslipidemia, thyroid disease, or others), and did not take medications affecting BP, glucose, or lipid levels or related to weight control. Among the initial participants, those who refused or missed measurements of body composition (19 females and 5 males), metabolic risk factors (185 females and 430 males), and CRF (17 females and 52 males) were excluded. Consequently, a total of 1183 university students (29.6% female) were included in the final data analyses (Figure 1). Sample size was determined based on the outcomes of our pilot study. Using an expected correlation coefficient of 0.11, we calculated that a sample size of 775 would be required at an α level of 0.05 and β level of 0.80, with the assumption of approximately 20% dropout rate (G*Power v3.1). The appropriate institutional review board reviewed and approved the study (approval no. SKKU 2017-06-011-001) in accordance with the Declaration of Helsinki. All participants provided written informed consent.

### 2.2. Variables

#### 2.2.1. Assessment of Anthropometrics and Definition of Metabolic Syndrome

Height (m) and weight (kg) were assessed with a stadiometer (Jenix, Seoul, South Korea), and body mass index (BMI) was calculated in unit of kg/m^2^. Body fat (%) was measured with the X-scan impedance body fat analyzer (Jawon Medical Co., Kyungsan, South Korea) according to the American College of Sports Medicine (ACSM) guidelines [20]. Waist circumference (WC) measurements were taken at the midpoint between the iliac crest and the lowest rib. BP measurement was conducted with an automated BP machine (Jawon Medical Co., Kyungsan, South Korea). The BP measurement was duplicated with a five-minute interval, and the mean values were used. Blood samples were taken from an antecubital vein after an 8- to 10-h fast. Fasting blood glucose, total cholesterol (TC), TG, and HDLC were assessed with an Ektachem DT-60 II analyzer (Johnson & Johnson Clinical Diagnostics, Inc., Rochester, NY, USA).

According to the Adult Treatment Program III of the National Cholesterol Education Program (NCEP ATP II) [21] with a Korean-specific definition of WC [22], the presence of Mets was defined as having three or more of the following attributes: (i) elevated WC of ≥85 cm in women or ≥90 cm in men; (ii) elevated TG of ≥150 mg/dL, (iii) elevated resting BP of ≥130/85 mmHg or taking BP-controlling medications, (iv) reduced HDLC of <50 mg/dL in women or <40 mg/dL in men, and (v) elevated fasting glucose of ≥100 mg/dL or taking glucose-lowering medications.

#### 2.2.2. Questionnaire

Eating speed was assessed by five possible responses, namely, very slow, slow, average, fast, and very fast, to the question “how fast do you eat relative to others?” Then, “very fast” and “fast” were combined into the eating quickly category, while “very slow,” “slow,” and “average” were combined into the eating slow category [12]. The validity and reliability of the questionnaire were previously tested and reported [23]. Health behaviors such as smoking (categorical: current/past or never), alcohol consumption (categorical: yes or no), and physical activity (categorical: yes or no) were assessed. Physical activity was assessed with the response to the question “how often (days per week) do you participate in moderate and/or vigorous physical activity?” Physical inactivity was defined as less than two days of moderate and/or vigorous physical activity per week.

#### 2.2.3. Measurement of Cardiorespiratory Fitness

Maximal oxygen uptake (VO_2_max), as the gold standard indicator of CRF, was measured with graded exercise testing on a motor-driven treadmill (Quinton TM55 Treadmill, Cardiac Science Corporation, Bothell, WA, USA) according to the ACSM guidelines [20]. Criteria for reaching VO_2_max was determined by at least two of the following four criteria being met: (1) a leveling off of VO_2_, (2) rate of perceived exertion (RPE) greater than 17 for the original category scale, (3) volitional exertion, and (4) reaching the age-predicted maximal heart rate. Subjects were verbally encouraged to exercise to the point of exhaustion during the test. Consequently, all subjects tested to exhaustion, and 95% of them achieved at least two of the four criteria. CRF was dichotomized as fit (upper 75th percentile) or unfit (lower 25th percentile) based on sex-specific individual values.

### 2.3. Statistics

Outcomes are presented as mean ± standard deviation (SD) values or percentages. Pearson’s chi-squared test and analysis of variance were used to compare categorical and continuous variables, respectively. An absence of multicollinearity among the three exposures was assessed with variance inflation factors (VIFs). VIF values were 1.017 for eating speed, 1.013 for nighttime snacking, 1.038 for physical activity, and 1.036 for CRF. Logistic regression analysis was conducted to estimate odds ratios (ORs) and 95% confidence intervals (CIs) of eating speed (quickly vs. slow), physical activity (inactive vs. active), and CRF (unfit vs. fit) for the onset of Mets. Statistical significance was tested at *p* = 0.05 with the Statistical Package for the Social Sciences for Windows version 27.0 software program (IBM Corporation, Armonk, NY, USA).

## 3. Results

Table 1 describes the study participants. Male students were older (*p* < 0.001) and heavier (*p* < 0.001); had higher rates of smoking (*p* < 0.001) and alcohol consumption (*p* < 0.001); and higher CRF (*p* < 0.001), higher WC (*p* < 0.001), higher resting BP (*p* < 0.001), higher FBG (*p* < 0.001), and higher TG (*p* < 0.001) in conjunction with a lower percentage of body fat (*p* < 0.001) and lower HDLC (*p* < 0.001) than female students. The prevalence of Mets was 8.8% in total; 11.1% among males and 3.4% among females (*p* < 0.001 for sex).

Table 2 presents descriptive statistics of study participants by self-reported eating speed, physical activity, and CRF. Students with fast eating habits were older (*p* < 0.001) and had a higher rate of smoking (*p* < 0.001), higher BMI (*p* < 0.001), higher percent body fat (*p* < 0.050), higher WHR (*p* < 0.001), higher WC (*p* < 0.001), higher SBP (*p* < 0.001), higher DBP (*p* < 0.050), higher TG (*p* < 0.001), and higher prevalence of Mets (*p* < 0.050) in conjunction with lower HDLC (*p* < 0.001) than students with slow eating habits. Inactive students had a higher percentage of body fat (*p* < 0.050), higher TG (*p* < 0.050), and higher prevalence of Mets (*p* < 0.001) than active students. Finally, unfit students were older (*p* < 0.001) and had higher BMI (*p* < 0.001), higher WC (*p* < 0.001), higher WHR (*p* < 0.001), higher SBP (*p* < 0.001), higher DBP (*p* < 0.01), higher FBG (*p* < 0.050), higher TG (*p* < 0.001), and higher prevalence of Mets (*p* < 0.001) than fit students.

Table 3 presents the relative risks of Mets by the three risk factors. Eating quickly was associated with higher risks for Mets (OR = 1.794; 95% CI = 1.196–2.691; *p* = 0.005), abdominal obesity (OR = 2.708; 95% CI = 1.916–3.826; *p* < 0.001), elevated TG (OR = 1.989; 95% CI = 1.426–2.773; *p* < 0.001), and reduced HDLC (OR = 1.381; 95% CI = 1.077–1.771; *p* = 0.011). The ORs for abdominal obesity (*p* < 0.001), elevated TG (*p* = 0.024), and reduced HDLC (*p* = 0.012) remained statistically significant even after adjustments for age, sex, smoking, and alcohol consumption. Physical inactivity was associated with higher risks for Mets (OR = 2.403; 95% CI = 1.503–3.841; *p* < 0.001), abdominal obesity (OR = 1.846; 95% CI = 1.268–2.687; *p* = 0.001), elevated BP (OR = 2.319; 95% CI = 0.939–1.216; *p* = 0.015), elevated FBG (OR = 1.281; 95% CI = 0.999–1.643; *p* = 0.050), elevated TG (OR = 1.470; 95% CI = 1.033–2.091; *p* = 0.032), and reduced HDLC (OR = 1.310; 95% CI = 1.022–1.678; *p* = 0.033). The ORs for Mets (*p* = 0.001), abdominal obesity (*p* = 0.004), and elevated BP (*p* = 0.024) remained statistically significant even after adjustments for age, sex, smoking, and alcohol consumption. Finally, lower CRF was significantly associated with higher risks for Mets (OR = 6.316; 95% CI = 4.133–9.652; *p* < 0.001), abdominal obesity (OR = 7.444; 95% CI = 5.143–9.652; *p* < 0.001), elevated BP (OR = 3.273; 95% CI = 1.799–5.955; *p* < 0.001), elevated FBG (OR = 1.450; 95% CI = 1.087–1.935; *p* = 0.011), and elevated TG (OR = 3.783; 95% CI = 2.650–5.399; *p* < 0.001) compared to higher CRF. The ORs for Mets (*p* < 0.001), abdominal obesity (*p* < 0.001), elevated BP (*p* = 0.001), elevated FBG (*p* = 0.032), and elevated TG (*p* < 0.001) remained statistically significant even after adjustments for age, sex, smoking, and alcohol consumption.

Table 4 presents the outcomes of the multivariate logistic regression analysis for Mets. A higher risk for Mets was significantly associated with fast eating (OR = 1.666; 95% CI = 1.094–2.601; *p* = 0.040), physical inactivity (OR = 1.687; 95% CI = 1.024–2.708; *p* = 0.018), and poor CRF (OR = 5.378; 95% CI = 3.475–8.325; *p* < 0.001). The strength of the association between the modifiable risk factors and Mets existed in the order of poor CRF (β = 1.682), fast eating (β = 0.523), and physical inactivity (β = 0.510).

## 4. Discussion

In this cross-sectional study involving 1891 Korean university students, we examined the relationship of lifestyle risk factors, such as fast eating, physical inactivity, and poor CRF, with Mets. We found that the lifestyle risk factors are significantly associated with Mets in Korean university students. Novel to our investigation was the observation that all three lifestyle risk factors are independent risk factors of Mets to be monitored in young adults.

In accordance with the current findings of this study, some evidence linking physical inactivity and poor CRF to Mets risk among university students from Western [8,24,25,26] and Asian countries [27,28,29] already exists in the literature. In particular, López-Martínez et al. [24] found that vigorous physical activity, CRF, and muscle strength were inversely associated with Mets risk in 275 Spanish university students aged 18 to 30 years. In another sample of 784 Columbian university students aged 20.1 ± 2.6 years, García-Hermoso et al. [30] also reported that male walking commuters were at lower risk for obesity, elevated BP, and decreased HDLC compared with their passive counterparts. In a population-based study consisting of 1078,685 Swedish male adolescents aged 16 to 19 years, Henriksson et al. [31] found that CRF was an independent predictor of later risks for cerebrovascular disease, ischemic heart disease, and heart failure.

Likewise, evidence reporting the prognostic role of physical activity and fitness in relation to Mets is also available among Korean university students. In a sample of 185 Korean university students, for example, Kim et al. [28] showed that fitness was negatively associated with BMI, WC, fasting glucose, and DBP, while BMI was positively associated with WC, TG, and SBP, implying a combined impact of fitness and BMI on the onset of Mets. In a web-based survey involving 2201 Korean university students, Ahn et al. [29] reported that physical inactivity was significantly associated with various physical symptoms or illnesses by comparing least active individuals with most active individuals. In a secondary data analysis of the 2010–2014 Korean National Health and Nutrition Examination Survey involving 1310 Korean university students, Jang and Kim [32] found that the prevalence of Mets was 5.3% in total, 6.5% in men and 4.1% in women, and concluded that smoking, frequent drinking, and lack of exercise were major behavioral risk factors of Mets. In our study, the prevalence of Mets was 8.8% in the total, 11.1% among male students, and 3.4% among female students, implying its rising prevalence, especially among male students.

Several explanations are viable regarding the beneficial effects of physical activity and CRF against Mets. They include (i) increased insulin sensitivity via increased glucose transporters [33]; (ii) better glycemic control via pancreatic β-cell insulin secretory compensation [34]; (iii) enhanced mitochondrial biogenesis [35]; (iv) improved lipoprotein lipids, such as decreased TG and increased HDLC, via enhanced activity of lipoprotein lipase [36,37]; (v) enhanced endothelial-derived NO production together with endothelial function [38,39]; (vi) suppressed inflammatory responses in conjunction with enhanced anti-inflammatory responses [40,41]; and (vii) healthier body composition [42,43].

With respect to eating speed, the current findings are in agreement with previous research reporting that eating quickly is positively associated with hypertriglyceridemia [15], overweight [12] or obesity [18], gestational diabetes [44], and glucose intolerance [14]. In a three-year follow study involving 1314 Japanese university students (*n* = 638 women), Yamane et al. [13] showed that eating quickly was significantly associated with an increased risk of being overweight. In a nationwide study involving 50,037 children aged 7 to 17 years, Zeng et al. [45] found that eating quickly was positively correlated with general obesity, abdominal obesity, and a greater waist-to-hip ratio, while eating slowly was negatively associated with general obesity, abdominal obesity, and a greater waist-to-hip ratio. As a plausible explanation, eating slowly may lead to less satiety and less fullness by altering feeding or satiety endocrinology [46]. Consequently, it is likely that those who eat more quickly consume more food than those who eat at a slower pace [19] or vice versa [18]. Together, the current findings suggest that eating speed should be considered as another risk factor of Mets to be monitored and intervened among university students in Korea.

This study has limitations. First, any causal explanation regarding the current findings cannot be given due to the cross-sectional nature of the study. Second, behavioral risk factors for Mets may have different patterns between men and women [47,48]. In this study, however, we did not conduct sex-specific data analysis due to (1) low statistical power related to a relatively small sample size of female students and (2) chance of type 2 error (or false negative). Sex difference in the association between Mets and risk factors remains to be further investigated in Korean university students.

## 5. Conclusions

Due to Westernized lifestyles in concert with physical inactivity and poor fitness, it is likely that the burden of metabolic disorders in the future will increase among Korean university students. Early detection of behavioral risk factors may provide useful information for preparing strategies to delay the onset of Mets and its associated health complications, which often become chronic. From a public health perspective, therefore, the current findings of the study suggest that a multifaceted intervention with physical activity, CRF promotion, and healthy eating behaviors should be implemented as a low-cost, safe measure against Mets in Korean university students.

## Figures and Tables

**Table 1:** Description of study participants.

Variables	Total (*n* = 1183)	Females (*n* = 351)	Males (*n* = 832)	*p* Value
Age (years)	23.2 ± 2.6	21.4 ± 2.0	24.0 ± 2.4	<0.001
BMI (kg/m^2^)	22.2 ± 3.0	20.4 ± 2.5	23.0 ± 2.8	<0.001
Body fat (%)	21.2 ± 5.4	24.5 ± 4.3	19.7 ± 5.2	<0.001
WHR	0.83 ± 0.05	0.80 ± 0.05	0.85 ± 0.05	<0.001
WC (cm)	79.8 ± 8.1	74.3 ± 6.5	82.1 ± 7.6	<0.001
VO_2_max (mL/kg/min)	43.4 ± 8.8	36.0 ± 5.5	46.6 ± 8.0	<0.001
Smoking, *n* (%)	415 (35.1)	27 (7.7)	388 (46.6)	<0.001
Alcohol intake, *n* (%)	884 (74.7)	199 (56.7)	685 (82.3)	<0.001
SBP (mmHg)	116.6 ± 13.5	106.3 ± 10.3	121.0 ± 12.2	<0.001
DBP (mmHg)	68.5 ± 8.6	64.1 ± 7.4	70.3 ± 8.5	<0.001
FBG (mg/dL)	97.0 ± 12.8	93.7 ± 11.5	98.3 ± 13.1	<0.001
TG (mg/dL)	103.1 ± 52.1	82.4 ± 31.4	111.9 ± 56.4	<0.001
HDLC (mg/dL)	48.8 ± 14.5	55.7 ± 14.7	45.9 ± 13.4	<0.001
Mets (*n*, %)	104 (8.8)	12 (3.4)	92 (11.1)	<0.001

BMI: body mass index; WHR: waist-to-hip ratio; WC: waist circumference; VO_2_max: maximal oxygen consumption; SBP: systolic blood pressure; DBP: diastolic blood pressure; FBG: fasting blood glucose; TG: triglycerides; HDLC: high-density lipoprotein cholesterol; Mets: metabolic syndrome.

**Table 2:** Descriptive statistics of study participants by eating speed, physical activity, and cardiorespiratory fitness.

Variable	Eating Speed		Physical Activity		Cardiorespiratory Fitness	
	Slow	Fast	Active	Inactive	Fit	Unfit
Age (years)	23.0 ± 2.5	23.6 ± 2.6 **	23.2 ± 2.6	23.2 ± 2.5	23.1 ± 2.5	23.8 ± 2.6 **
BMI (kg/m^2^)	21.7 ± 2.8	23.3 ± 3.0 **	22.4 ± 2.6	22.1 ± 3.2	21.8 ± 2.6	24.0 ± 3.8 **
Body fat (%)	20.9 ± 5.5	21.6 ± 5.2 *	20.6 ± 5.1	21.2 ± 5.6 *	20.3 ± 5.2	24.2 ± 5.0 **
WHR	0.83 ± 0.05	0.85 ± 0.05 **	0.83 ± 0.05	0.83 ± 0.05	0.83 ± 0.05	0.86 ± 0.05 **
WC (cm)	78.4 ± 7.7	82.5 ± 8.2 **	79.8 ± 7.3	79.9 ± 8.7	78.4 ± 7.0	85.0 ± 9.9 **
Current/past smokers (*n*, %)	236 (30.4)	179 (44.0) **	185 (37.4)	230 (66.6)	289 (32.5)	107 (44.0)
Alcohol intake (*n*, %)	568 (73.2)	316 (77.6)	369 (74.7)	515 (74.7)	654 (73.5)	189 (77.8)
SBP (mmHg)	115.1 ± 13.3	119.5 ± 13.4 **	117.4 ± 13.2	116.1 ± 13.7	115.5 ± 12.9	120.7 ± 15.3 **
DBP (mmHg)	67.9 ± 8.5	69.5 ± 8.8 *	68.1 ± 7.9	68.8 ± 9.1	67.8 ± 8.1	70.9 ± 10.1 **
FBG (mg/dL)	96.9 ± 13.2	97.1 ± 12.0	96.3 ± 11.7	97.4 ± 13.5	96.3 ± 12.4	98.9 ± 13.3 *
TG (mg/dL)	98.1 ± 47.9	112.7 ± 58.0 **	99.4 ± 48.2	105.8 ± 54.5 *	95.6 ± 43.3	130.0 ± 65.7 **
HDLC (mg/dL)	50.1 ± 14.8	46.2 ± 14.5 **	49.1 ± 14.4	48.5 ± 14.5	49.1 ± 14.4	47.3 ± 14.8
Metabolic syndrome (*n*, %)	55 (7.1)	49 (12.0) *	27 (5.5)	77 (11.2) **	43 (4.8)	59 (24.3) **

BMI: body mass index; WHR: waist-to-hip ratio, WC: waist circumference; SBP: systolic blood pressure; DBP: diastolic blood pressure; FBG: fasting blood glucose; TG: triglycerides; HDLC: high-density lipoprotein cholesterol. Asterisks (* and **) indicate a statistical significance (*p* = 0.05 and *p* = 0.001, respectively).

**Table 3:** Odds ratios and 95% confidence intervals for metabolic syndrome and its components according to risk factors in univariate logistic regression.

Variables	Eating Quickly		Inactive		Unfit	
	Crude OR (95% CI)	Adjusted OR (95% CI)	Crude OR (95% CI)	Adjusted OR (95% CI)	Crude OR (95% CI)	Adjusted OR (95% CI)
Mets	1.794 * (1.196–2.691)	1.416 (0.928–2.160)	2.403 ** (1.503–3.841)	2.176 * (1.381–3.429)	6.316 ** (4.133–9.652)	5.500 ** (3.546–8.528)
Abdominal obesity	2.708 ** (1.916–3.826)	2.292 ** (1.600–3.282)	1.846 * (1.268–2.687)	1.710 * (1.187–2.464)	7.444 ** (5.143–10.775)	6.855 ** (4.680–10.041)
Elevated BP	1.717 (0.955–3.084)	1.370 (0.751–2.499)	2.319 * (0.939–1.216)	2.150 * (1.104–4.185)	3.273 ** (1.799–5.955)	2.808 * (1.498–5.265)
Elevated FBG	0.953 (0.743–1.222)	0.785 (0.604–1.020)	1.281 * (0.999–1.643)	1.217 (0.956–1.548)	1.450 * (1.087–1.935)	1.378 * (1.022–1.859)
Elevated TG	1.989 ** (1.426–2.773)	1.486 * (1.053–2.098)	1.470 * (1.033–2.091)	1.301 (0.925–1.828)	3.783 ** (2.650–5.399)	3.533 ** (2.426–5.144)
Reduced HDLC	1.381 * (1.077–1.771)	1.391 * (1.074–1.800)	1.310 * (1.022–1.678)	1.273 (0.997–1.625)	1.274 (0.952–1.706)	1.253 (0.931–1.688)

OR: odds ratio; CI: confidence interval; BP: blood pressure; FBG: fasting blood glucose; TG: triglycerides; HDLC: high-density lipoprotein cholesterol. Adjusted for age, sex, smoking, and alcohol consumption. Asterisks (* and **) indicate statistical significance (*p* = 0.05 and *p* = 0.001, respectively).

**Table 4:** Odds ratios and 95% confidence intervals for metabolic syndrome according to risk factors in a multivariate logistic regression.

Variables		β	Chi-Square	Adjusted OR (95% CI)	*p* Value
Eating quickly	No			Reference	
	Yes	0.523	5.591	1.687 (1.094–2.601)	0.018
Physical activity	Active			Reference	
	Inactive	0.510	4.231	1.666 (1.024–2.708)	0.040
Physical fitness	Fit			Reference	
	Unfit	1.682	56.981	5.378 (3.475–8.325)	<0.001

OR: odds ratio; CI: confidence interval. OR adjusted for age, sex, smoking, and alcohol consumption.

## Data Availability

Data can be accessible upon request to corresponding author (hkang@skku.edu).
